# Supplementary material for: Organized primary human papillomavirus–based cervical screening: A randomized healthcare policy trial
Source: PLoS Med. 2021 Aug 23;18(8):e1003748. doi: 10.1371/journal.pmed.1003748 (PMC8423359; doi:10.1371/journal.pmed.1003748)
Supplement: Attachment — Submitted filename: Elfstrom_et_al_Response_20210626.docx [file pmed.1003748.s003.docx]

Dear Dr. Louise Gaynor-Brook,

Thank you for the invitation to revise and re-submit our manuscript "Effectiveness of organized primary HPV-based cervical screening: randomized healthcare policy".

We have revised the paper in accordance with the comments and the revisions made are detailed point-by-point below. We hope that the revisions will be considered appropriate, and that the manuscript will now meet with your acceptance.

Kind regards and on behalf of all authors,

Joakim Dillner

-----------------------------------------------------------

**Requests from the editors:**

Data availability:

PLOS Medicine requires that the de-identified data underlying the specific results in a published article be made available, without restrictions on access, in a public repository or as Supporting Information at the time of article publication, provided it is legal and ethical to do so. Please note that, if the data are only freely available upon request, a study author cannot be the contact person for the data. Please provide an alternative, independent contact for data requests, and their web or email address.

*The data access has been clarified. The data is deposited with the Swedish Cervical Screening Registry (*[*www.nkcx.se_en*](http://www.nkcx.se_en)*) and can be requested at* [*info@nkcx.se*](mailto:info@nkcx.se) *(administrator Sara Nordquist Kleppe).*

Title: Please revise your title according to PLOS Medicine's style. Please place the study design in the subtitle (ie, after a colon). We suggest “Organized primary human papillomavirus-based cervical screening: a randomized healthcare policy trial” or similar

*The title has been modified accordingly.*

Abstract:

Please report your abstract according to CONSORT for abstracts, following the PLOS Medicine abstract structure (Background, Methods and Findings, Conclusions) <http://www.consort-statement.org/extensions?ContentWidgetId=562> taking into account the extra guidance for non-inferiority trials (<https://jamanetwork.com/journals/jama/fullarticle/1487502>)

Abstract Background: Provide expand on the background to your study and the context of why the study is important. The final sentence should clearly state the study question.

Abstract Methods and Findings:

Please provide brief demographic details of the study population (e.g. age, ethnicity, etc)

Please provide the number in each group.

Please include the study design, and the dates during which study enrollment and follow up occurred

In the last sentence of the Abstract Methods and Findings section, please describe 2-3 of the main limitation(s) of the study's methodology.

Please begin your Abstract Conclusions with "In this study, we observed ..." to briefly summarise the main findings of your study. Please address the study implications, mentioning only specific implications substantiated by the results.

*These changes have been made, except on ethnicity (registration of ethnicity is not considered ethical in Sweden).*

Author Summary:

At this stage, we ask that you include a short, non-technical Author Summary of your research to make findings accessible to a wide audience that includes both scientists and non-scientists. The Author Summary should immediately follow the Abstract in your revised manuscript. This text is subject to editorial change and should be distinct from the scientific abstract. Please see our author guidelines for more information: <https://journals.plos.org/plosmedicine/s/revising-your-manuscript#loc-author-summary>

In the final bullet point of ‘What Do These Findings Mean?’, please describe the main limitation(s) of the study in non-technical language.

*These additions have been made.*

Introduction:

Please address past research (have there been any other studies reporting on implementation of HPV-based screening within RHPs?) and explain the potential importance of your study.

Line 66 - please define RCT at first use

Please conclude the Introduction with a clear description of the study question or hypothesis (framed more as a question than ‘we report on ‘).

*These textual additions and edits have been made.*

Methods:

Please include the study protocol document and analysis plan, with any amendments, as Supporting Information to be published with the manuscript if accepted.

Thank you for providing a CONSORT checklist. Please revise this to use section and paragraph numbers, rather than page numbers, and ensure to take into account the extra guidance for non-inferiority trials.

Please add the following statement, or similar, to the Methods: "This study is reported as per the Consolidated Standards of Reporting Trials (CONSORT) guideline (S1 Checklist)."

*These changes have been made and the original study protocol from 2011 including the analysis plan is now included.*

Line 99 - please define ASCUS and LSIL at first use

Line 140 - please define HSIL at first use

Line 157 - Please replace "subject" with participant, individual, or person.

*These changes have been made.*

Results:

Please provide a table (Table 1) showing the baseline characteristics of the study population.

The sample size listed in the submitted manuscript and the trial registry differ. Please explain the discrepancy.

*Baseline characteristics have been added to Tables 1a and 1b. The sample size listed in the trial registry was calculated based on actual enrollment, not women invited. In the present paper, we also describe women invited to screening as this is required by the CONSORT format. Furthermore, a pilot implementation included in the original protocol which has been separately published (Lamin et al., BMJ Open 2014).*

Paragraph 2 - please specify what the numbers in brackets represent

Lines 206 / 209 / 218 - please specify the comparison group

Line 210 - please omit ‘both’

Line 222 - please clarify in which group ‘detection of invasive cancers was somewhat lower’

Line 231 - please clarify which result corresponds to which group

*These clarifications have been made.*

Discussion:

Please present and organize the Discussion as follows: a short, clear summary of the article's findings; what the study adds to existing research and where and why the results may differ from previous research; strengths and limitations of the study; implications and next steps for research, clinical practice, and/or public policy; one-paragraph conclusion.

Please remove all subheadings within your Discussion e.g. Limitations and other considerations

Line 301 - please correct reference call out in superscript

Line 312 - please temper assertions of primacy by adding ‘to the best of our knowledge’ or similar

*The discussion has been reformatted and the textual edits have been made.*

Tables:

In each table, when a p value is given, please specify the statistical test used to determine it.

Please define abbreviations used in the table legend of each table.

Please present numerators and denominators for percentages in each table.

*These edits to the tables have been made.*

References:

Please ensure that journal name abbreviations match those found in the National Center for Biotechnology Information (NCBI) databases, and are appropriately formatted and capitalised e.g. refs 7, 11, etc. Please also see <https://journals.plos.org/plosmedicine/s/submission-guidelines#loc-references> for further details on reference formatting.

*The references have been reformatted accordingly.*

**Comments from the reviewers:**

Reviewer #1: I confine my remarks to statistical aspects of this paper. These were very well done (and clearly explained) and I have only a couple of very minor requests before I can recommend publication.

*Thank you for this supportive comment.*

p. 5 Line 104-105 Please give a citation for this and, perhaps, some reasoning behind it. It seems counter-intuitive.

*This has been clarified and a reference added.*

p. 8 Line 163-164 Please give a citation for the Farrington-Manning score, and describe it, as it is relatively little known.

*A citation and description have been added.*

Peter Flom

Reviewer #2: The present manuscript by Elfström et al. deals with a topic that most experts in public health would agree is very important, namely, supplementing studies of efficacy with studies of effectiveness before implementing or significantly modifying interventional programme affecting large numbers of people.

The present authors have conducted a landmark study in which evidence-based modifications to improve a long-running, population-based cervical cancer screening programme have been evaluated in a trial involving randomization of all of the over 200,000 women invited to attend the programme over a 4-year period.

The results of the presently reported registered randomized public health policy trial verify that under real-life conditions the programme fulfilled the expectations of policymakers in modifying the programme.

This achievement is likely to receive wide attention by professionals and policymakers in public health and in women's health. The paper not only provides a model for future efforts to implement and modify large scale programmes.

The paper is also highly relevant, even though HPV vaccination in younger women has been universally recommended in the past decade for primary prevention of cervical cancer - because for older women who have already been infected by carcinogenic HPV organized cervical cancer screening will continue to be the most effective option for cervical cancer control for many years. Moreover, for younger women screening programmes will need to be adapted to monitor and evaluate vaccination programmes, and the landmark study of Elfström et al. provides a valuable example of how such modifications can be evaluated.

In addition to the topic, the methodology of the study, the organisation of the paper and the presentation and discussion of the results are exemplary and are likely to be emulated by researchers in the coming years.

I therefore recommend the paper for publication without modification.

*Thank you for this affirmation of our work.*

Reviewer #3: The manuscript is much clearer now. I have no mayor comments. I have spotted two very minor typos:

Page 21 line 206, there is an extra parenthesis '(45.80%'

Table 4. There is a 2 superscript after CIN2+ which does not have a footnote.

*Thank you. We have corrected the two typos.*

Reviewer #4: The authors describe: Effectiveness of organized primary HPV-based cervical screening: randomized healthcare policy.

This is indeed a large randomized study in comparing HPV based cervical cancer screening with cytology based screening.

However several issues need to be addressed in my opinion.

1. It is not clear to me whether women had to consent to this study, and if they were aware of randomisation and different assessment policies in the lab?? If no consent was obtained, explain why this was left out of the procedure.
   - *We revised the text to clarify these issues. Briefly, in Sweden the ethical Review Boards are appointed by government, chaired by a senior judge and have the authority to decide on consent requirements. In our case, the ERB decided that the act of participating after receiving information was to be regarded as consent. The information letters were open, i.e. they described randomization and the screening test assigned.*
2. Randomisation was done on the last digit of the PIN. But a large difference between size of the groups as well as age and attendence was found. Was this difference also there at time of invitation and if so why was it not corrected during the study to create equal groups at least at invitation.
   - *Indeed, we do not know why this imbalance in randomization occurred. But we have extensively checked the possible implications of this imbalance and the results are extensively documented. We do not consider it good scientific practice to repeat randomizations until a desirable result is obtained, thus the original randomization was adhered to.*
3. The groups consist of women with new as well as repeat invitations, and also on this aspect the groups differ. I would prefer to have groups as homogeneous as possible and would like to see an analysis of the new invitations only as especially repeat invitations of non-responders may cause more bias.
   - *We have run a sensitivity analysis on the main analyses of the paper (Table 4) restricted to new invitations only per suggested. As this analysis was not originally specified in the protocol, we have instead done it post-hoc at the suggestion of the reviewer. We have reported it briefly and descriptively in the results section.*
4. The analysis is done with data gathered in 2017. Given the 3 year interval for recall in case of HPV pos cytology negative or Low grade cytology HPV neg I would like to have those data incorporated as it will provide a much more reliable picture. We expect especially in the HPV positive cytology negative group a substantial number of CIN 2+, as other studies show that HPV screening with a single cytology triage misses a substantial number of cases.
   - *Although we agree that this would indeed have been very interesting, it takes a substantial amount of time to collect and analyze data from a cohort of half a million women (at least a year) and this data is therefore better to report in a follow-up paper, planned for 2023.*
5. Biopsies are used as histology result in this study. The inter and even intra observer variability is quite large especially on biopsies. this needs to be commented on in the discussion.
   - *Although histopathology is acknowledged as the “gold standard” in the field, the variability can be substantial. We now acknowledge this and discuss the possible effects in discussion.*
6. The referrel rate went up with an extra 0.36% in the HPV arm. This is much less as experienced in other countries. For instance in the netherlands referrals more than doubled after introduction of HPV based screening. On the other hand a 3.5% referral rate in the cytology arm is high compared to what we are used to, especially since the second triage step is not included here. Please comment on this.
   - *Indeed, “HPV-based screening” is a concept that can include several different referral and follow-up strategies. We have commented on this and discuss why we see only a limited increase compared to others.*
7. The most recent reference is of early 2019. Several important studies on this subject have been published since then, for instance Polman et al. Lancet Oncology, 20(2), 229-238, also a RCT in a screening population. so references must be updated and incorporated in the discussion.
   - *We have added both the Polman et al. reference as well as XXX to the discussion.*
