# Supplementary material for: Organized primary human papillomavirus–based cervical screening: A randomized healthcare policy trial
Source: PLoS Med. 2021 Aug 23;18(8):e1003748. doi: 10.1371/journal.pmed.1003748 (PMC8423359; doi:10.1371/journal.pmed.1003748)
Supplement: Attachment — Submitted filename: Elfsrom_et_al_Reviewer_Response_20210726.docx [file pmed.1003748.s004.docx]

Dear Dr. Louise Gaynor-Brook,

Thank you for the positive review and invitation to address editorial and production issues on our manuscript "Organized primary human papillomavirus-based cervical screening: a randomized healthcare policy trial".

We have revised the paper in accordance with the comments and the revisions made are detailed point-by-point below. We hope that the revisions will be considered appropriate, and that the manuscript will now meet with your acceptance.

Kind regards and on behalf of all authors,

Joakim Dillner

Dear Dr. Dillner,

Thank you very much for re-submitting your manuscript "Organized primary human papillomavirus-based cervical screening: a randomized healthcare policy trial" (PMEDICINE-D-21-00811R2) for review by PLOS Medicine.

I have discussed the paper with my colleagues and the academic editor and it was also seen again by one reviewer. I am pleased to say that provided the remaining editorial and production issues are dealt with we are planning to accept the paper for publication in the journal.

The remaining issues that need to be addressed are listed at the end of this email. Any accompanying reviewer attachments can be seen via the link below. Please take these into account before resubmitting your manuscript:

In revising the manuscript for further consideration here, please ensure you address the specific points made by each reviewer and the editors. In your rebuttal letter you should indicate your response to the reviewers' and editors' comments and the changes you have made in the manuscript. Please submit a clean version of the paper as the main article file. A version with changes marked must also be uploaded as a marked up manuscript file.

Please also check the guidelines for revised papers at <https://eur01.safelinks.protection.outlook.com/?url=http%3A%2F%2Fjournals.plos.org%2Fplosmedicine%2Fs%2Frevising-your-manuscript&amp;data=04%7C01%7Cjoakim.dillner%40ki.se%7C023ffb684c1a4e6a7b3908d94c52af3e%7Cbff7eef1cf4b4f32be3da1dda043c05d%7C0%7C0%7C637624740279012065%7CUnknown%7CTWFpbGZsb3d8eyJWIjoiMC4wLjAwMDAiLCJQIjoiV2luMzIiLCJBTiI6Ik1haWwiLCJXVCI6Mn0%3D%7C1000&amp;sdata=s7Dh5PRkMxtR%2F6M48hIFcVd%2Btuef3YeHr5kbiZAU3b8%3D&amp;reserved=0> for any that apply to your paper. If you haven't already, we ask that you provide a short, non-technical Author Summary of your research to make findings accessible to a wide audience that includes both scientists and non-scientists. The Author Summary should immediately follow the Abstract in your revised manuscript. This text is subject to editorial change and should be distinct from the scientific abstract.

We expect to receive your revised manuscript within 1 week. Please email us ([plosmedicine@plos.org](mailto:plosmedicine@plos.org)) if you have any questions or concerns.

We ask every co-author listed on the manuscript to fill in a contributing author statement. If any of the co-authors have not filled in the statement, we will remind them to do so when the paper is revised. If all statements are not completed in a timely fashion this could hold up the re-review process. Should there be a problem getting one of your co-authors to fill in a statement we will be in contact. YOU MUST NOT ADD OR REMOVE AUTHORS UNLESS YOU HAVE ALERTED THE EDITOR HANDLING THE MANUSCRIPT TO THE CHANGE AND THEY SPECIFICALLY HAVE AGREED TO IT.

Please ensure that the paper adheres to the PLOS Data Availability Policy (see <https://eur01.safelinks.protection.outlook.com/?url=http%3A%2F%2Fjournals.plos.org%2Fplosmedicine%2Fs%2Fdata-availability&amp;data=04%7C01%7Cjoakim.dillner%40ki.se%7C023ffb684c1a4e6a7b3908d94c52af3e%7Cbff7eef1cf4b4f32be3da1dda043c05d%7C0%7C0%7C637624740279012065%7CUnknown%7CTWFpbGZsb3d8eyJWIjoiMC4wLjAwMDAiLCJQIjoiV2luMzIiLCJBTiI6Ik1haWwiLCJXVCI6Mn0%3D%7C1000&amp;sdata=5OhbvwYaUrYT3nM8FYp35iFUVYoa96htKtdoFps0nmc%3D&amp;reserved=0>), which requires that all data underlying the study's findings be provided in a repository or as Supporting Information. For data residing with a third party, authors are required to provide instructions with contact information for obtaining the data. PLOS journals do not allow statements supported by "data not shown" or "unpublished results." For such statements, authors must provide supporting data or cite public sources that include it.

To enhance the reproducibility of your results, we recommend that you deposit your laboratory protocols in protocols.io, where a protocol can be assigned its own identifier (DOI) such that it can be cited independently in the future. Additionally, PLOS ONE offers an option to publish peer-reviewed clinical study protocols. Read more information on sharing protocols at <https://eur01.safelinks.protection.outlook.com/?url=https%3A%2F%2Fplos.org%2Fprotocols%3Futm_medium%3Deditorial-email%26utm_source%3Dauthorletters%26utm_campaign%3Dprotocols&amp;data=04%7C01%7Cjoakim.dillner%40ki.se%7C023ffb684c1a4e6a7b3908d94c52af3e%7Cbff7eef1cf4b4f32be3da1dda043c05d%7C0%7C0%7C637624740279012065%7CUnknown%7CTWFpbGZsb3d8eyJWIjoiMC4wLjAwMDAiLCJQIjoiV2luMzIiLCJBTiI6Ik1haWwiLCJXVCI6Mn0%3D%7C1000&amp;sdata=UbmW6gAIdPgbNOiXgmOytSj%2BMyRYSUtEdPXceoG%2FYPc%3D&amp;reserved=0>

Please review your reference list to ensure that it is complete and correct. If you have cited papers that have been retracted, please include the rationale for doing so in the manuscript text, or remove these references and replace them with relevant current references. Any changes to the reference list should be mentioned in the rebuttal letter that accompanies your revised manuscript.

Please note, when your manuscript is accepted, an uncorrected proof of your manuscript will be published online ahead of the final version, unless you've already opted out via the online submission form. If, for any reason, you do not want an earlier version of your manuscript published online or are unsure if you have already indicated as such, please let the journal staff know immediately at [plosmedicine@plos.org](mailto:plosmedicine@plos.org).

If you have any questions in the meantime, please contact me or the journal staff on [plosmedicine@plos.org](mailto:plosmedicine@plos.org).  

We look forward to receiving the revised manuscript by Jul 28 2021 11:59PM.   

Sincerely,

Louise Gaynor-Brook, MBBS PhD
Associate Editor 
PLOS Medicine
plosmedicine.org

------------------------------------------------------------
Requests from Editors:

Throughout the paper, please adapt reference call-outs to the following style: "... low-grade cervical cytological abnormalities [6,7]" (noting the absence of spaces within the square brackets).

*These edits have been made.*

Please revise your Author Summary, which should include 2-3 bullet points per subheading, in accessible and non-technical language. Please see e.g. <https://eur01.safelinks.protection.outlook.com/?url=https%3A%2F%2Fjournals.plos.org%2Fplosmedicine%2Farticle%3Fid%3D10.1371%2Fjournal.pmed.1003686&amp;data=04%7C01%7Cjoakim.dillner%40ki.se%7C023ffb684c1a4e6a7b3908d94c52af3e%7Cbff7eef1cf4b4f32be3da1dda043c05d%7C0%7C0%7C637624740279012065%7CUnknown%7CTWFpbGZsb3d8eyJWIjoiMC4wLjAwMDAiLCJQIjoiV2luMzIiLCJBTiI6Ik1haWwiLCJXVCI6Mn0%3D%7C1000&amp;sdata=ZjjX%2FrUYp3jtakZVgB9XQ7n0asps82U62GVEMIHYdjk%3D&amp;reserved=0> for a recent example. In the final bullet point of ‘What Do These Findings Mean?’, please describe the main limitation(s) of the study.

*The Author Summary has been revised.*

Introduction:
Line 110 - please revise beginning of sentence beginning ‘The purpose being…’

*This sentence has been revised.*

Methods:
Please refer to your study protocol / analysis plan early in the Methods section.

*We have now referred to the study protocol/analysis plan in the Methods section.*

Results:
Line 258 - please clarify ‘The proportion of positives that were positive…’

*This sentence has been clarified.*

Please add a final one-paragraph conclusion to your Discussion.

*A final concluding paragraph has been added to the Discussion.*

References:
Please ensure that journal name abbreviations match those found in the National Center for Biotechnology Information (NCBI) databases, and are appropriately formatted and capitalised.
Please also see <https://eur01.safelinks.protection.outlook.com/?url=https%3A%2F%2Fjournals.plos.org%2Fplosmedicine%2Fs%2Fsubmission-guidelines%23loc-references&amp;data=04%7C01%7Cjoakim.dillner%40ki.se%7C023ffb684c1a4e6a7b3908d94c52af3e%7Cbff7eef1cf4b4f32be3da1dda043c05d%7C0%7C0%7C637624740279012065%7CUnknown%7CTWFpbGZsb3d8eyJWIjoiMC4wLjAwMDAiLCJQIjoiV2luMzIiLCJBTiI6Ik1haWwiLCJXVCI6Mn0%3D%7C1000&amp;sdata=O9VET46OithdzB2I3gVDizoJIOCMo0pwKWdwpTBezeo%3D&amp;reserved=0> for further details on reference formatting. E.g. Ref 7 should be Contemp Clin Trials, ref 11 should be BMJ Open
There appears to be additional unnecessary information in ref 17

*The abbreviations in the reference list have been reviewed and corrected.*

Comments from Reviewers:

Reviewer #4: the authors have addressed all issues raised very well. I recommend acceptance in its present form.

*Thank you for this feedback.*
